# Supplementary material for: A cholinergic-sympathetic pathway primes immunity in hypertension and mediates brain-to-spleen communication
Source: Nat Commun. 2016 Sep 27;7:13035. doi: 10.1038/ncomms13035 (PMC5052663; doi:10.1038/ncomms13035)
Supplement: Supplementary Information — Supplementary Figures 1-9. [file ncomms13035-s1.pdf]

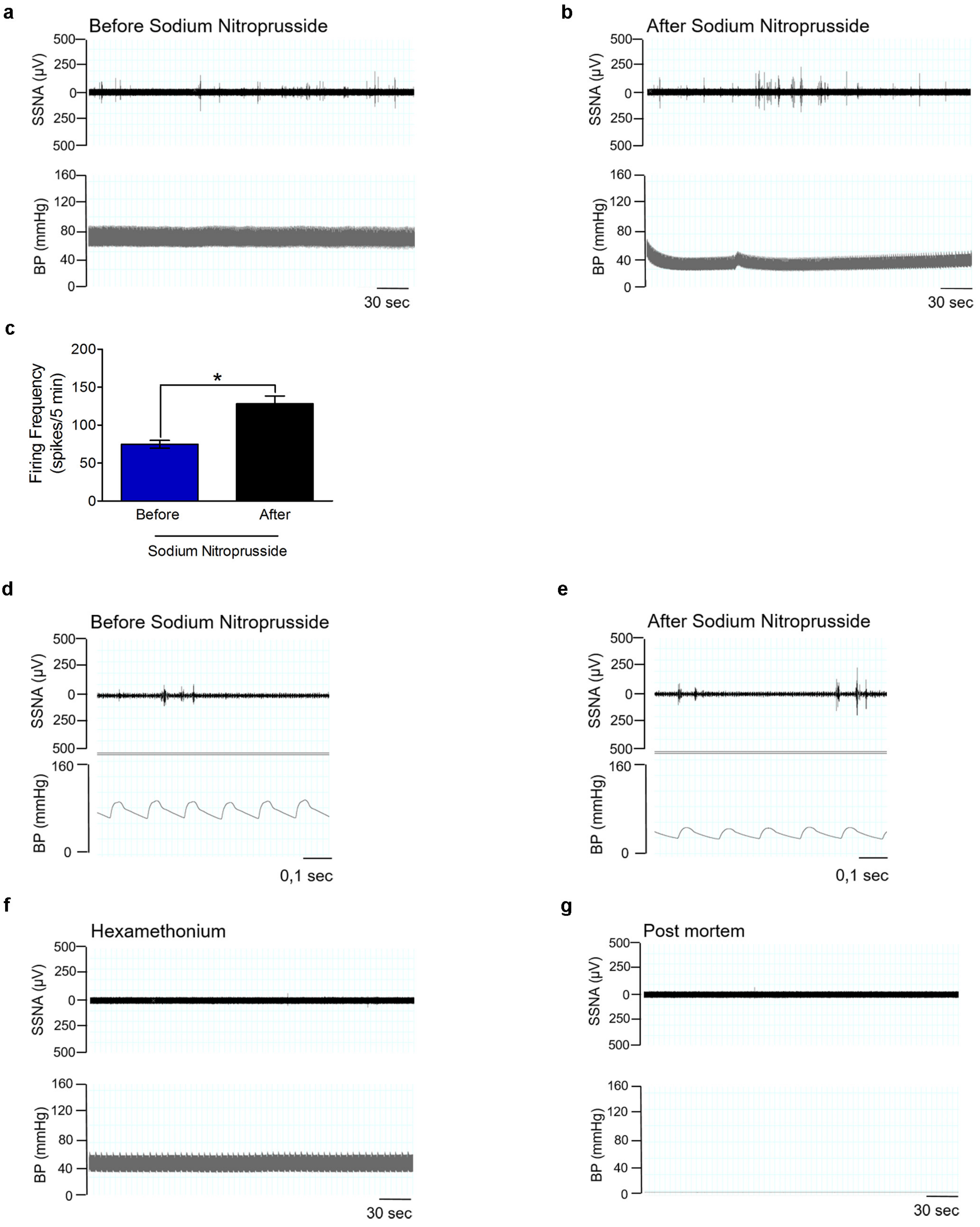

Supplementary Figure 1. Acute fall in blood pressure determines activation of sympathetic nerve activity in splenic nerve. (**a**) Representative recording of splenic sympathetic nerve activity (SSNA) during baseline (upper panel) and corresponding raw blood pressure signal (lower panel). (**b**) Effect of Sodium Nitroprusside (SNP) administration on SSNA activity (upper panel) and blood pressure (lower panel). (**c**) Quantitative analysis of firing frequency indicating a significant increase in SSNA after SNP-induced fall in blood pressure (n_{mice}=4; Paired Samples Student's t-test, t(3) = 5.442, *p<0.05). (**d,e**) Representative details of recordings showing that sympathetic burst are pulse synchronous, after the peak of Systolic Blood Pressure. (**f,g**) Representative recording of SSNA activity (upper panel) and blood pressure (lower panel) obtained after ganglionic blockade with Hexamethonium (**f**) and postmortem (**g**).

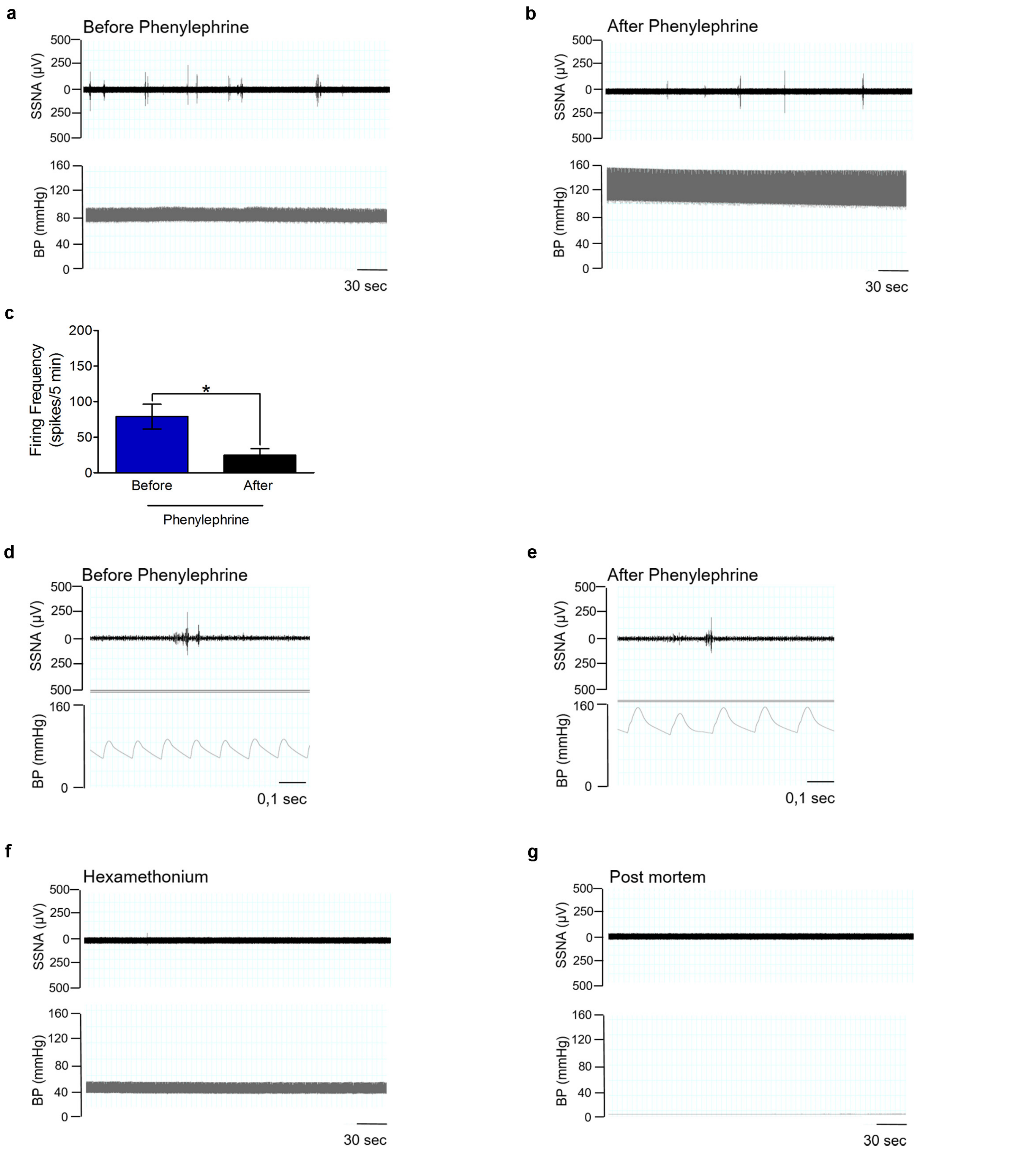

Supplementary Figure 2. Acute increase in blood pressure inhibits activation of sympathetic nerve activity in splenic nerve. (**a**) Representative recording of splenic sympathetic nerve activity (SSNA) during baseline (upper panel) and corresponding raw blood pressure signal (lower panel). (**b**) Effect of Phenylephrine (Phe) administration on SSNA activity (upper panel) and blood pressure (lower panel). (**c**) Quantitative analysis of firing frequency confirming the significant inhibitory effect of blood pressure increase on SSNA (n_{mice}=4;Paired Samples Student's t-test, t(3) = 4.035, *p<0.05). (**d,e**) Representative details of recordings showing that sympathetic burst are pulse synchronous, after the peak of Systolic Blood Pressure. (**f,g**) Representative recording of SSNA activity (upper panel) and blood pressure (lower panel) obtained after ganglionic blockade with Hexamethonium (**f**) and postmortem (**g**).

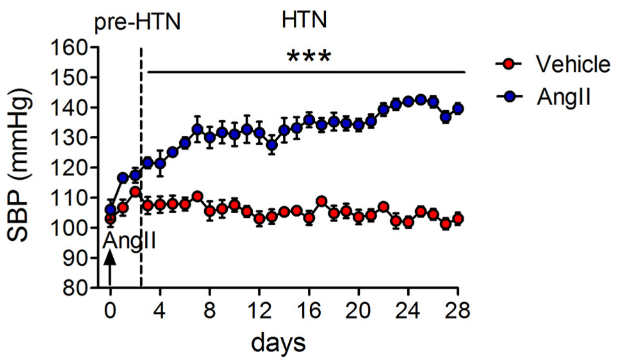

Supplementary Figure 3. Three days of AngII infusion is the earliest time point for blood pressure increase. Blood pressure measurement in mice infused with AngII or Vehicle for 28 days indicates that three days is not yet significantly different from that of control mice (Veh and AngII n_{mice}=7 for; Two-way ANOVA for repeated measures; Systolic Blood Pressure SBP, $F_{(interaction)}$= 10.96, ***p<0.001).

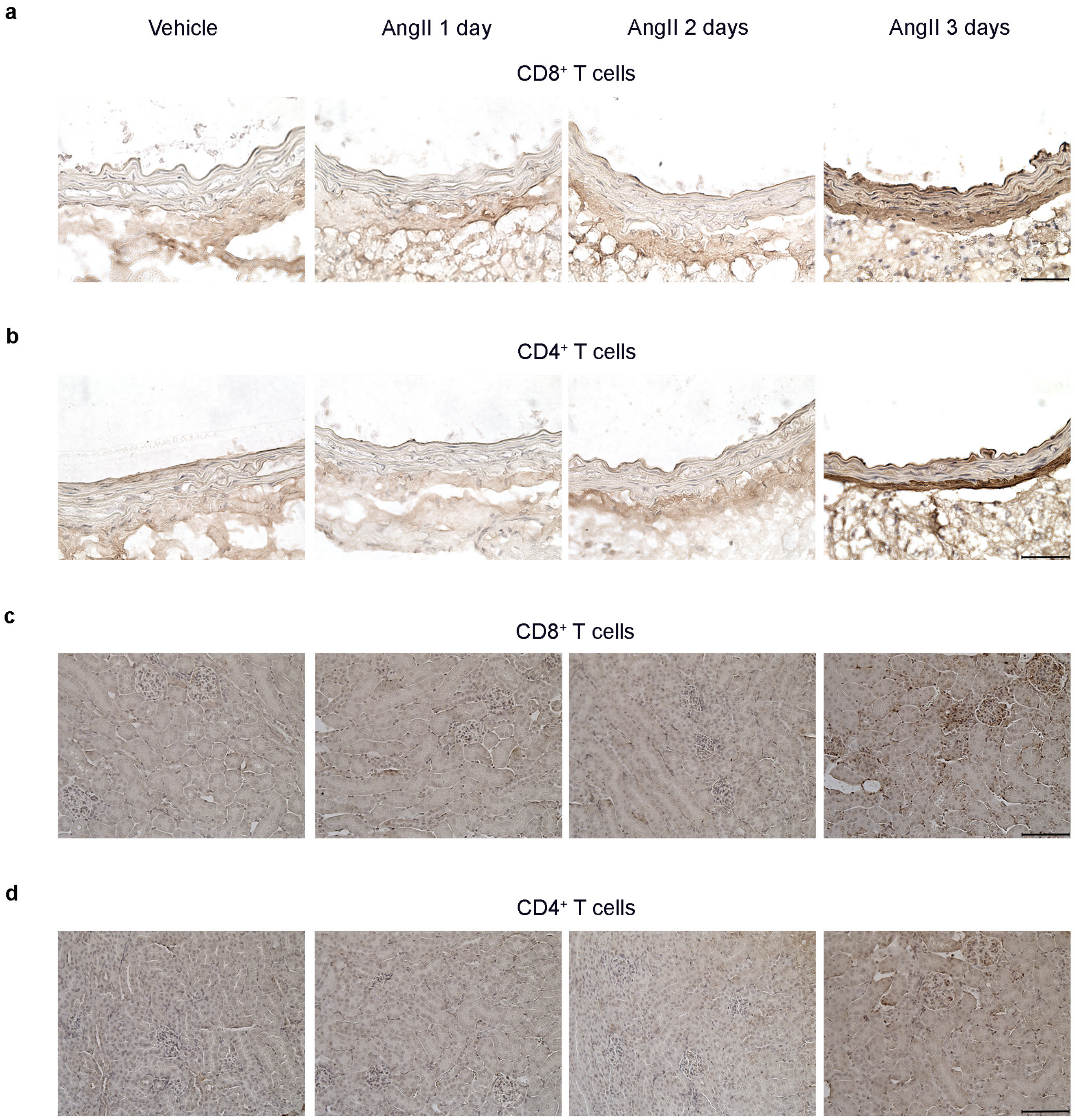

Supplementary Figure 4. Three days of AngII infusion is the earliest time point for immune system activation. (a-d) Analysis of $CD8^{+}$ and $CD4^{+}$ T cells in aortas (**a**,**b**) and kidneys (**c**,**d**) of mouse infused with Vehicle or AngII for 1, 2 and 3 days shows that the early infiltration is visible at 3 days (n_{mice}=6 for each group). Scale bar (**a**,**b**), 50 μm and (**c**,**d**) 100 μm.

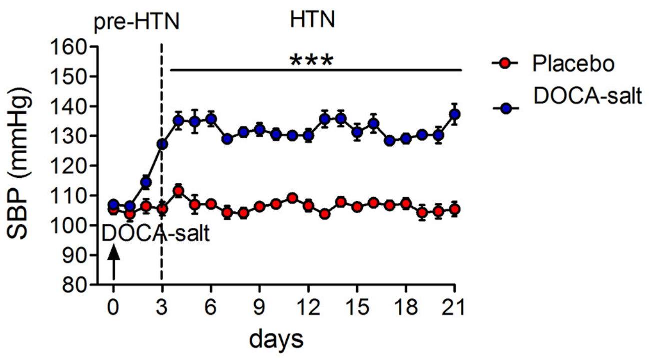

Supplementary Figure 5. DOCA-salt challenge induces hypertension in WT mice. DOCA-salt rises blood pressure in a time-dependent way, with a significant steady-state increase starting at 3 days after pellet implantation (Placebo and DOCA-salt n_{mice}=7; Two-way ANOVA for repeated measures; Systolic Blood Pressure SBP, $F_{(interaction)}$= 7.784, ***p<0.001).

a

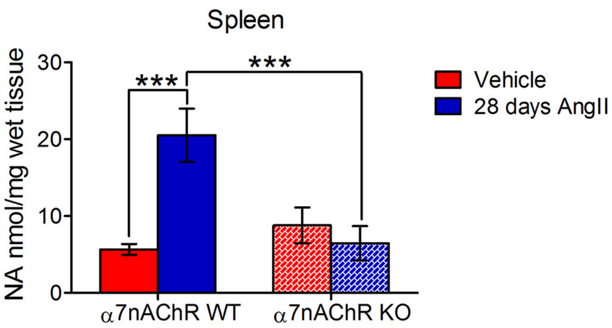

b

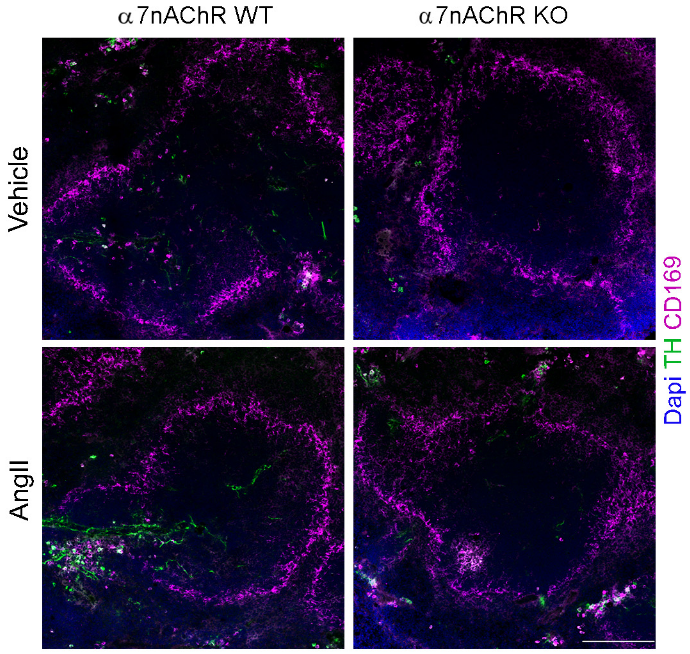

Supplementary Figure 6. AngII fails to increase noradrenaline in the spleen in α7nAChR KO. (**a**) Analysis of noradrenaline content in the spleen further indicates that AngII fails to synthetize the neurotransmitter in α7nAChR KO mice (n_{mice}=6 for each group; Two-way ANOVA, $F_{(interaction)}$ = 12.88, ***p<0.001). (**b**) Tyrosine hydroxylase staining (green) shows that α7nAChR KO mice are protected from the activation typically observed in the splenic marginal zone, delineated by $CD169^{+}$ cells (magenta), in WT mice upon AngII (n_{mice}=6 for each group; scale bar, 200 μm).

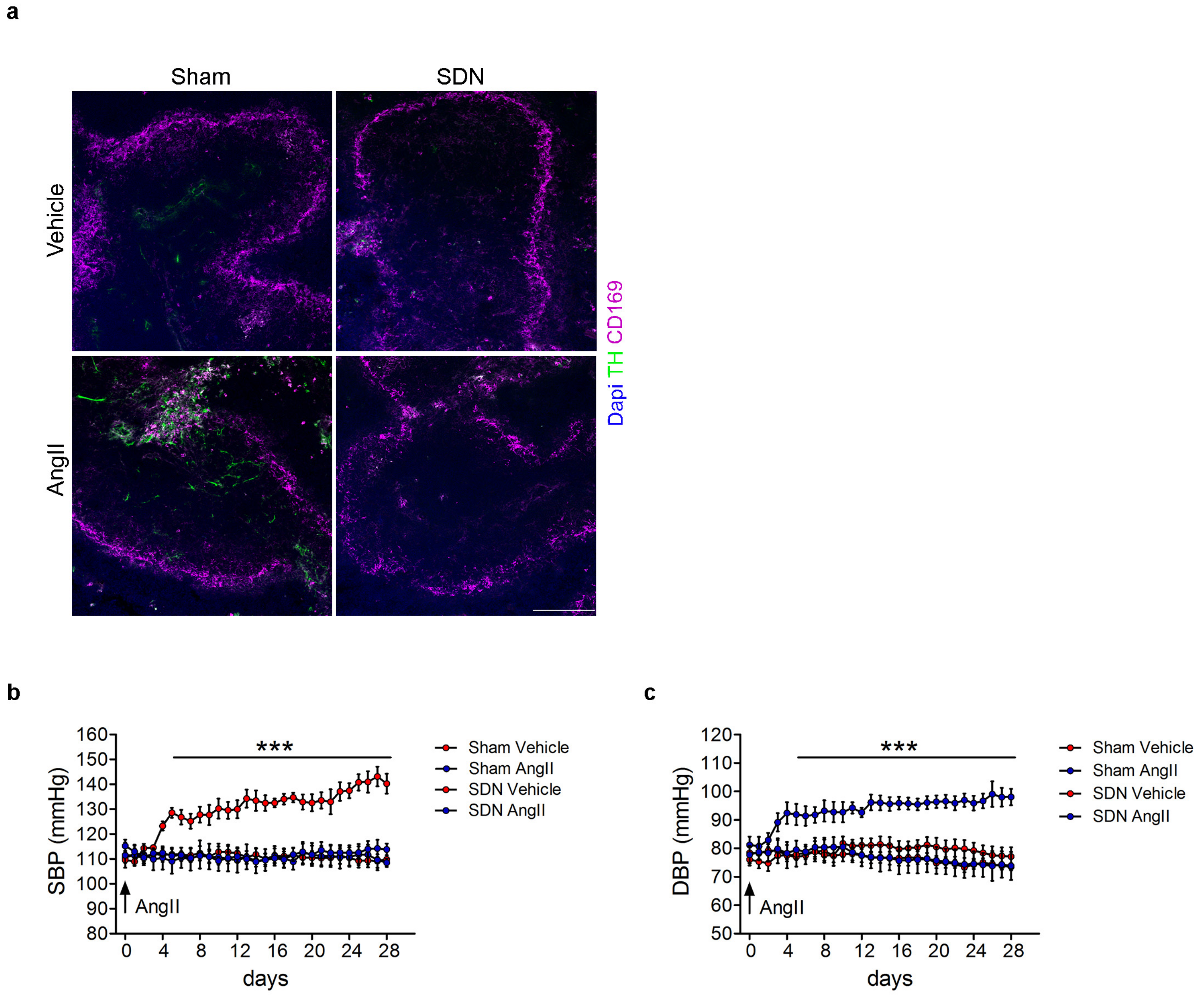

Supplementary Figure 7. Splenic denervation protects from the activation of noradrenaline synthesis and blood pressure raising induced by AngII. (**a**) A representative immunofluorescence of tyrosine hydroxylase staining (green) and $CD169^{+}$ cells (magenta), delineating the splenic marginal zone, shows the loss of sympathetic fibers in mice subjected to splenic denervation as compared to Sham (n_{mice}=6 for each group; scale bar, 200 μm). (**b,c**) Radiotelemetric blood pressure measurement further indicating that splenic denervation protects mice from AngII induced hypertension (n_{mice}=6 for each group; Two-way ANOVA, (**b**) Systolic Blood Pressure SBP $F_{(interaction)} = 11.436$, ***$p<0.001$; (**c**) Diastolic Blood Pressure DBP $F_{(interaction)} = 7.217$, **$p<0.01$ and ***$p<0.001$).

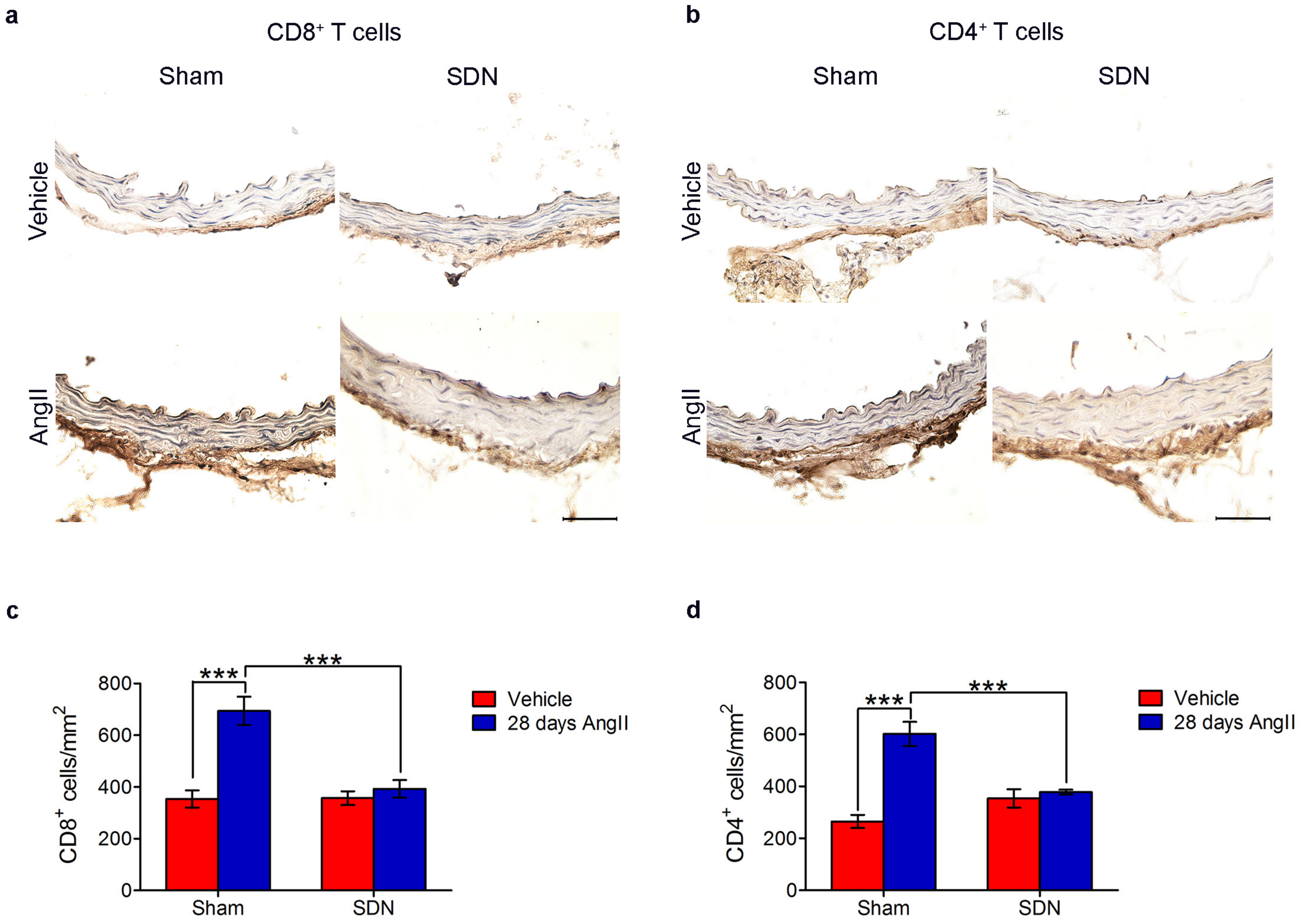

Supplementary Figure 8. Splenic denervation protects from the T cells infiltration typically observed in aortas of mice infused with AngII for 28 days, as revealed by immunohistochemistry. (**a-d**) Representative immunohistochemistry (**a**,**b**) and quantitative analysis (**c**,**d**) of $CD8^+$ and $CD4^+$ T cells in SDN and sham mice infused with AngII or Vehicle for 28 days (n_{mice}=6 for each group; Two-way ANOVA, CD8 $F_{(interaction)}$ = 15.40, ***p<0.001; CD4 $F_{(interaction)}$ = 23.79, ***p<0.001). Scale bar, 50 μ m.

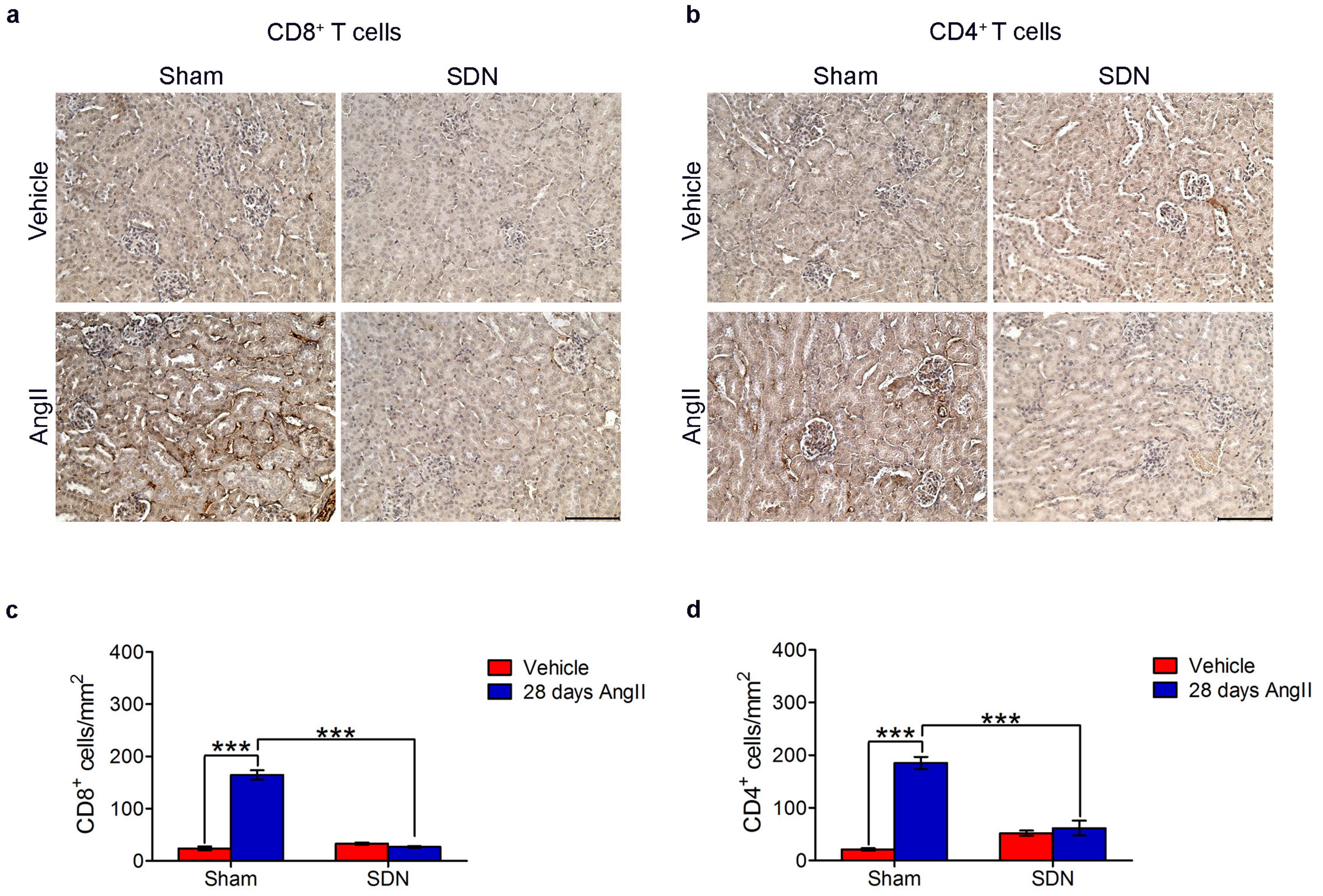

Supplementary Figure 9. Splenic denervation protected kidneys from infiltration of T cells induced by chronic AngII, as revealed by immunohistochemistry. (**a-d**) Representative immunohistochemistry (**a**,**b**) and quantitative analysis (**c**,**d**) of $CD8^+$ and $CD4^+$ T cells in SDN and sham mice infused with AngII or Vehicle for 28 days (n_{mice}=6 for each group; Two-way ANOVA, CD8 $F_{(interaction)}$ = 225.4, ***p<0.001; CD4 $F_{(interaction)}$ = 65.01, ***p<0.001). Scale bar, 100 μm.
